# *Escherichia coli* O157 Cluster Evaluation

**DOI:** 10.3201/eid1010.040374

**Published:** 2004-10

**Authors:** Amita Gupta, Susan B. Hunter, Sally A. Bidol, Stephen Dietrich, Jennifer Kincaid, Ellen Salehi, Lisa Nicholson, Carol Ann Genese, Sarah Todd-Weinstein, Lisa Marengo, Akiko C. Kimura, John T. Brooks

**Affiliations:** * Centers for Disease Control and Prevention, Atlanta, Georgia, USA;; † Michigan Department of Community Health, Lansing, Michigan, USA:; ‡ Ohio Department of Health, Columbus, Ohio, USA;; § New Jersey Department of Health and Senior Services, Trenton, New Jersey, USA;; ¶ New York State Department of Health, Albany, New York, USA;; # Texas Department of Health, Austin, Texas;; ** California Department of Health Services, Sacramento, California, USA

**Keywords:** Escherichia coli O157:H7, pulsed-field gel electrophoresis, PFGE, United States, dispatch

## Abstract

We investigated a multistate cluster of *Escherichia coli* O157:H7 isolates; pulsed-field gel electrophoresis subtyping, using a single enzyme, suggested an epidemiologic association. An investigation and additional subtyping, however, did not support the association. Confirmating *E. coli* O157 clusters with two or more restriction endonucleases is necessary before public health resources are allocated to follow-up investigations.

*Escherichia coli* O157:H7 is an important cause of foodborne infections estimated to cause 73,000 illnesses and 60 deaths annually in the United States (*1*). Implementation of pulsed-field gel electrophoresis (PFGE) molecular subtyping has greatly improved *E. coli* O157:H7 surveillance and detection of outbreaks (*2*). PFGE subtyping was initially used to identify related isolates and support epidemiologic associations during outbreak investigations. Public health laboratories in the United States now routinely subtype all *E. coli* O157:H7 isolates by PFGE as part of a national molecular subtyping network (PulseNet) (*2*) after this practice proved instrumental in identifying outbreaks not detected by traditional epidemiologic methods (*3*). PulseNet laboratories initially digest isolates with a single enzyme and compare the resulting PFGE patterns by using commercial software (BioNumerics, St. Martens-Latem, Belgium) to determine whether patterns are shared by multiple isolates. These patterns are then communicated electronically to the Centers for Disease Control and Prevention (CDC) (Atlanta, GA), where PFGE patterns of isolates from different states are definitively compared. PulseNet policy states that isolates with potential epidemiologic significance that have indistinguishable patterns with a primary enzyme, should be digested with a secondary enzyme before extensive epidemiologic investigations are undertaken. Indistinguishable patterns should also be confirmed by submission to a central database (*2*). However, time constraints and the availability of sufficient resources prevent some laboratories from adhering to this policy.

## The Study

On July 5, 2000, the Michigan Department of Community Health's laboratory notified CDC of a cluster of five *E. coli* O157:H7 isolates, collected from May 25 to June 21, 2000, which shared an indistinguishable *Xba*I PFGE pattern. PulseNet staff confirmed that these isolates' patterns were indistinguishable and designated the pattern as PulseNet pattern EXHX01.0047. In 2000, this PFGE pattern represented approximately 2% of the *E. coli* O157 patterns in the PulseNet database. These Michigan isolates possessed genes only for Shiga toxin 2 (*stx*2) but not Shiga toxin 1 (*stx*1); approximately 30% of *E. coli* O157 isolates sent to CDC since 1983 expressed only *stx*2. From July through September 2000, six states (California, Michigan, New Jersey, New York, Ohio, and Texas) reported a total of 64 *E. coli* O157 isolates with PulseNet pattern EXHX01.0047, a value that exceeded expectation for this time of year and that prompted an epidemiologic investigation. Not all of these patterns were submitted to CDC's central database for confirmation. Fifty-one of these isolates were probed for Shiga toxin genes, and all possessed only *stx*2. Illness onsets ranged from April 1 through August 21, 2000, with a notable increase after late May 2000. The median age of case-patients was 13 years (range 1–91), and 38 (60%) were female; 36 (57%) of 64 were hospitalized, and hemolytic uremic syndrome developed in 9 (14%).

To determine the source of these *E. coli* O157:H7 infections, six state health departments (California, Michigan, New Jersey, New York, Ohio, and Texas) and CDC initiated an epidemiologic investigation. Informed consent was obtained from all patients or their parents or guardians and human experimentation guidelines of the U.S. Department of Health and Human Services were followed. These data were collected as part of an outbreak investigation and therefore were exempt from formal institutional review boardaprovals.

Through hypothesis-generating interviews with 19 infected persons, 11 food exposures were reported by >50% of interviewees or were reported in substantial excess relative to that food's frequency of consumption in the general population (*4*). In a case-control study that used a survey instrument that focused on these 11 food exposures, controls were matched to case-patients by sex and age group, and were asked about exposures during the same 5-day period before the matching case-patient's illness onset. Controls were contacted and identified by using sequential-digit dialing beginning with the matching patient's telephone number.

Twenty-eight case-patients and 69 matched controls were enrolled (2.46 controls per patient). The median age was 13.5 years and 50% were female; case-patients did not differ significantly from controls in terms of sex or age. In matched univariate analysis by using logistic regression with stratification (LogXact version 2.1.1, Cytel Software Corporation, Cambridge, MA), only broccoli was significantly associated with illness (matched odds ratio [mOR] 3.65, p = 0.04); 14 (58%) of 24 case-patients, and 19 (31%) of 62 controls reported eating broccoli. Although none of the three foods that contained ground beef were individually associated with illness, consumption of "any hamburger" (a composite variable) was significant (mOR 7.30, p = 0.01), reported by 20 (87%) of 23 patients and 28 (55%) of 51 controls. In multivariate analysis, only eating "any hamburger" remained significantly associated with illness (OR = 6.13, p = 0.02).

Ground beef eaten by case-patients was recovered from three households; samples from two households (in New Jersey and California) yielded *E. coli* O157:H7 isolates that were indistinguishable from PulseNet pattern EXHX01.0047. One of these isolates was tested for Shiga toxin and produced only *stx*2. Using information from these two cases and additional information regarding likely ground beef sources for the original Michigan cases, the U.S. Department of Agriculture performed a traceback; however, an extensive investigation did not identify any common supplier for the two samples of ground beef.

**Figure 1:**
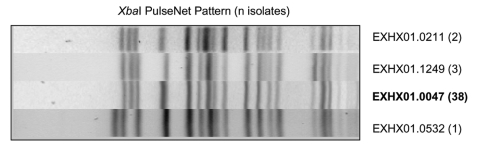
*Xba*I-generated pulsed-field gel electrophoresis patterns for *Escherihia coli* O157 isolates reported as indistinguishable from PulseNet pattern EXHX01.0047.

**Figure 2:**
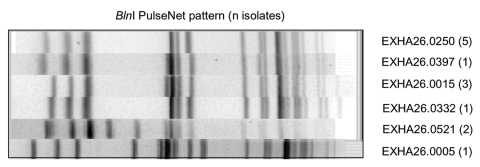
*Bln*I-generated pulsed-field gel electrophoresis patterns for *Esscherichia coli* O157 isolates that generated patterns indistinguishable from PulseNet pattern EXHX01.0047 by using *Xba*I.

We performed a retrospective review of available isolate patterns received by the PulseNet national database after the case-control study and traceback had been completed. Four additional states (Florida, Indiana, Massachusetts, and Washington) had reported *E. coli* O157 isolates with *Xba*I patterns that were indistinguishable from PulseNet pattern EXHX01.0047. Among the 46 submitted *Xba*I patterns from states reporting a possible match to PulseNet pattern EXHX01.0047, analysis at CDC indicated that 38 were indistinguishable and that 6 differed by one band from the PulseNet pattern EXHX01.0047 (Figure 1). Furthermore, among the 38 isolates confirmed as PulseNet pattern EXHX01.0047, digestion of 13 isolates with the restriction enzyme *Bln*I produced PFGE patterns that sorted into multiple distinct clusters (Figure 2).

## Conclusions

Identifying outbreaks of *E. coli* O157:H7 infections by routinely subtyping isolates using PFGE is a relatively new phenomenon (*2**,**3*). Traditionally, PFGE has been used to support or refute the likelihood of epidemiologic relatedness among case-patients and suspect food vehicles in epidemiologic investigations. In this instance, the converse occurred; the results of routine PFGE subtyping (*Xba*I) of *E. coli* O157:H7 isolates prompted a large, multistate epidemiologic investigation. Isolates were potentially related because 1) the PFGE patterns obtained with one restriction enzyme (*Xba*I) were reported to be indistinguishable and a relatively uncommon pattern, and 2) the isolates shared a Shiga toxin profile that was relatively uncommon among *E. coli* O157 (*stx*2 only). A rigorous case-control study implicated a widely consumed food vehicle responsible for multiple past outbreaks of *E. coli* O157 infections: ground beef (*5*). This study and the isolation of two *E. coli* O157 with matching PFGE patterns from ground beef consumed by case-patients prompted two extensive traceback investigations. However, no common source could be identified. Subsequent digestion of patient isolates with a second enzyme showed that they were actually part of multiple, small clusters and that the illnesses were thus unlikely to be related to a common source.

Investigation of suspected multistate outbreaks requires substantial public health resources (*6*). This investigation involved more than 50 federal, state, and local staff. *E. coli* O157:H7 infections can cause serious and potentially life-threatening illness that may also engender legal action. Public health authorities must ensure that linkage of illnesses to an outbreak be as complete and accurate as possible. Rapid identification of the infections' source can avert many potential illnesses. Earlier studies demonstrated the value of subtyping *E. coli* O157:H7 isolates with two or more restriction endonuclease digestions or using other subtyping methods, such as phage typing, to determine whether such isolates are truly related, even if these isolates have produced matching patterns using a single enzyme digestion (*7**–**9*). More recently, in the absence of epidemiologic data, single enzyme PFGE has been found to be a poor measure of genetic relatedness (*10*). Since 1998, the PulseNet Task Force has recommended the use of at least two enzyme digestions for optimal subtyping of *E. coli* O157 isolates. However, because of resource limitations, many state and local public health laboratories initially subtype *E. coli* O157 isolates with *Xba*I enzyme and perform subtyping with a second enzyme only if clusters are identified and personnel and resources are in place to do so. This investigation lends further support to the conclusion that when clusters of *E. coli* O157 are detected on the basis of subtyping data only (i.e., in the absence of any epidemiologic data), digestion with two or more endonucleases is warranted, even if the isolates appear to share a primary enzyme pattern or possess other microbiologic evidence of clonality (e.g., Shiga toxin profile). Furthermore, these findings underscore the importance of having a centralized database team that can rapidly verify reports of clusters from participating PulseNet laboratories and assist in determining whether isolates are likely to be part of an outbreak and whether a rapid, large-scale epidemiologic investigation and traceback would be warranted.
